# Frontiers and literature review on non-aspiration stroke-associated infections

**DOI:** 10.1186/s40001-026-03881-4

**Published:** 2026-01-21

**Authors:** Wen Zhao, Xuanyue Yu, Yuhua Zhang, Shuya Cai, Jianwen Lin, Pan Qin, Yi Sui, Shiwei Du, Dong Chen, Yi Liu

**Affiliations:** 1 Department of Neurology, Central Hospital of Dalian University of Technology, Dalian, 116033 China; 2 Department of Neurology, Jiangmen Central Hospital, Jiangmen, 529030 China; 3 Dalian Medical University, Dalian, 116021 China; 4 School of Control Science and Engineering, Dalian University of Technology, Dalian, 110091 China; 5 Department of Neurology and Neurointervention, Shenyang First People’s Hospital, Shenyang Medical College, Shenyang, 110122 China; 6 Department of Neurosurgery, Shenzhen University General Hospital, Shenzhen University Clinical Medical Academy, Shenzhen, 518055 China; 7 Department of Neurosurgery, Central Hospital of Dalian University of Technology, Dalian, 116033 China; 8 Institute of Cadio-Cerebrovascular Medicine, Dalian University of Technology, Dalian, 116033 China

**Keywords:** Non-aspiration, Stroke-associated infection, Risk factors, Pathophysiology

## Abstract

Stroke-associated infection (SAI) is a significant complication of stroke, with approximately 30% of stroke patients developing infections, primarily respiratory and urinary tract infections. These infections can severely impact prognosis and increase the economic burden. While dysphagia is a known independent risk factor for stroke-associated pneumonia, research shows that stroke patients without aspiration or related risk factors, such as dysphagia and impaired consciousness, also exhibit a high incidence of non-aspiration stroke-associated infections (NA-SAI). This comprehensive article meticulously examines and reviews the risk factors, pathophysiological underpinnings, and therapeutic strategies for NA-SAI, with the aim of enhancing clinicians' comprehension and management of this condition.

## Introduction

Stroke ranks among the leading causes of global mortality and disability. According to the Global Burden of Disease 2021 study, it results in 7.3 million annual deaths and over 160 million disability-adjusted life years (DALYs) [1]. Professor Budinčević et al. reported that the total 1-year cost per ischemic stroke patient in Croatia was €18,221, with an average quality-adjusted life year (QALY) of 0.372, underscoring the profound impact of stroke on quality of life [2]. In China, the incidence of stroke among individuals aged ≥ 40 years is rising annually [3], compounded by surging hospitalization costs, posing a critical public health challenge. While reperfusion therapies (e.g., intravenous thrombolysis/endovascular thrombectomy) improve acute outcomes in ischemic stroke, stroke-associated infection (SAI)—a core complication—is independently linked to adverse outcomes and mortality [4], significantly affecting patient recovery and quality of life. Notably, previous SAI research has primarily focused on aspiration mechanisms, but our team’s prior data demonstrate that NA-SAI accounts for over half of SAI cases [5]. However, the pathophysiology, risk factors, and management strategies for NA-SAI remain poorly understood. Urgent research is needed to address these gaps and ultimately enhance clinical care for stroke patients.

The latest Global Burden of Disease 2021 data indicate that ischemic stroke constitutes 65.3% of all new stroke cases [1], with regional variations but consistent predominance. Beyond its immediate life-threatening effects, acute ischemic stroke triggers acute and chronic complications, including SAI, hemorrhagic transformation, and seizures. The overall incidence of SAI ranges from 20 to 30%, with lower rates in high-income countries (18–25%) and higher rates in low- and middle-income countries (30–35%) [6]. In contrast, hemorrhagic transformation (0–85%) [7] and seizures (2–20%) [8] exhibit marked variability. Among these complications, SAI stands out due to its high incidence and regional disparities. SAI is defined as infections occurring within 7 days of stroke onset, excluding pre-existing or incubation-period infections, with respiratory and urinary tract infections being most common [9]. Recent advances in SAI research have revealed that non-aspiration stroke-associated infections also occur frequently in stroke patients. Furthermore, Barlas et al. [10] reported that non-aspiration pneumonia patients had significantly higher risks of in-hospital mortality, prolonged hospitalization, sepsis, respiratory failure, and seizures. Although this study did not directly focus on NA-SAI, it implies that NA-SAI may have distinct clinical characteristics and severe adverse outcomes, significantly impacting patient prognosis.

NA-SAI is defined as infections occurring within 7 days of acute stroke onset in patients without pre-existing infections or incubation-period infections, after excluding aspiration-related factors (e.g., dysphagia or altered consciousness). While specific incidence data for NA-SAI are currently lacking, its clinical significance cannot be understated. Our team’s preliminary research indicates that NA-SAI incidence exceeds aspiration-associated SAI, and existing studies highlight its unique clinical features and poor outcomes. However, research on the pathophysiology, risk factors, and preventive/therapeutic strategies for NA-SAI remains insufficient. A comprehensive understanding of NA-SAI is essential for addressing stroke complications holistically and developing effective management strategies.

### Pathophysiological mechanisms

**Fig. 1:**
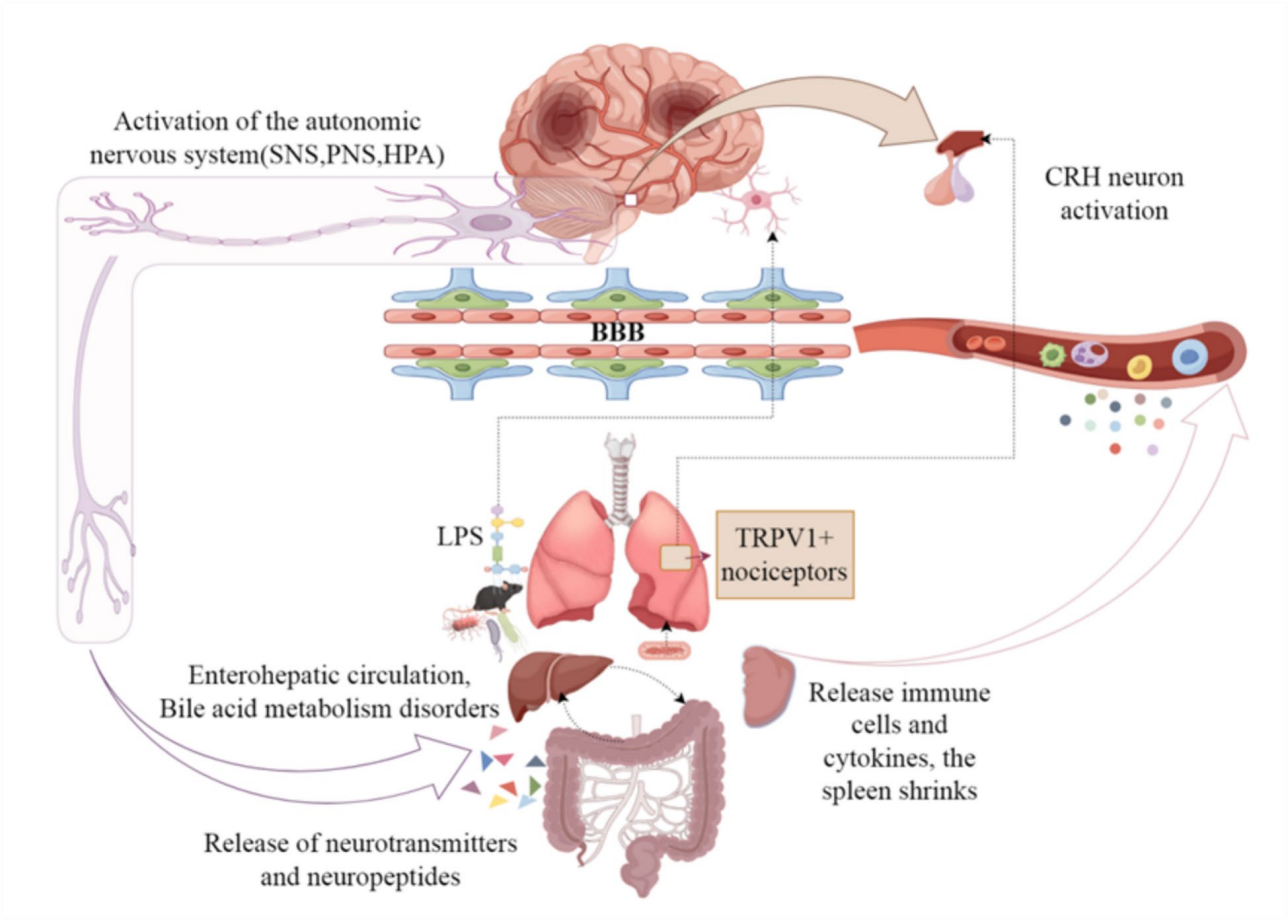
provides a comprehensive overview of the primary pathophysiological mechanisms underlying non-aspiration stroke-associated infections (NA-SAI). Following stroke onset, activation of the autonomic nervous system—including the sympathetic nervous system (SNS), parasympathetic nervous system (PNS), and the hypothalamic–pituitary–adrenal (HPA) axis—leads to immunosuppression. Additionally, the involvement of nociceptors expressing transient receptor potential vanilloid 1–positive (TRPV1⁺) cells, activation of corticotropin-releasing hormone (CRH) neurons, disruption of the blood–brain barrier (BBB), and the effects of lipopolysaccharide (LPS) collectively impact the lung–brain axis. The autonomic nervous system modulates the gut microbiota through the release of neurotransmitters and peptides, alters bile acid metabolism via the gut–liver circulation, and influences the release of immune cells and cytokines, as well as inducing splenic atrophy. These combined effects on the immune system and inflammatory responses ultimately increase the risk of NA-SAI

This paper provides a comprehensive summary of the pathophysiological mechanisms underlying NA-SAI, primarily encompassing post-stroke immunosuppression, immune inflammatory response, the Gut-Brain axis, and the Lung-Brain axis, as illustrated in Fig. 1.

#### Post-stroke immunosuppression

Stroke-Induced Immunosuppression Syndrome (SIISS): SIISS involves peripheral immune function changes post-stroke, including lymphocyte reduction, decreased monocyte activity, Th1 to Th2 cell conversion, changes in NK cell activity, and decreased IFN-γ production, all of which increase infection risk [11]. Early studies described T lymphocyte reduction post-stroke, with subsequent animal experiments highlighting the importance of systemic immunosuppression. Reduction in T, B, CD4 + T, CD8 + T, and NK cells, particularly CD4 + T cells, correlates with increased infection rates. SIISS is closely related to T cell subset activity balance conversion, particularly the Th1 to Th2 shift. Th1 cells produce pro-inflammatory cytokines, while Th2 cells produce anti-inflammatory or regulatory cytokines [12]. Other T cell types, such as regulatory T cells (Treg) and Th17 cells, further complicate the immune regulatory network. Liesz et al. found that increasing Treg numbers can reduce Th1 responses, promoting Th2-type cytokine production and providing protection [13].

Monocytes and macrophages participate in post-stroke immune regulation and repair processes through the conversion of pro-inflammatory (M1) and anti-inflammatory (M2) phenotypes [14]. Multiple mechanisms regulate M1/M2 conversion, involving inflammatory mediators like chemokines (e.g., interleukin-1β, tumor necrosis factor-α), are released, which can activate monocytes and macrophages, promoting their differentiation into pro-inflammatory M1 phenotype. Additionally, multiple transcription factors, such as NF-κB (nuclear factor kappa B), promote M1 formation, while STAT6 (signal transducer and activator of transcription-6) activation contributes to M2 development [15]. This regulation mechanism aims to maintain immune balance and repair damaged tissues, but it may lead to immune imbalance and inflammatory damage in certain situations. Moreover, monocyte HLA-DR (Human Leukocyte Antigen-DR) is a major histocompatibility complex class II molecule that plays a crucial role in antigen presentation and T cell activation; pathogen invasion stimulates the innate immune response [16]. Monocyte surface HLA-DR reflects the immune function state, and several studies indicate that bacterial infections and sepsis in critically ill patients are closely related to low HLA-DR expression on monocytes [17, 18]. Hoffmann et al. found that low monocyte HLA-DR expression is an independent risk factor for SAP (stroke-associated pneumonia) by collecting biomarkers from patients with acute ischemic stroke [19]. The downregulation of HLA-DR, coupled with the increase in immunosuppressive cell subsets and the inhibitory cytokines they produce, such as interleukin-10 and transforming growth factor-β, leads to immune function suppression and weakened inflammatory responses, which increases the risk of infection.

The interaction between the nervous system and the immune system during SIISS involves the sympathetic nervous system, the hypothalamic–pituitary–adrenal (HPA) axis, and the cholinergic vagus nerve.

##### Sympathetic nervous system

Studies on experimental stroke animal model show that excessive activation of the sympathetic nervous system plays an essential role in immunosuppression and the occurrence of stroke-associated pulmonary infections. These studies found that using the β-receptor blocker propranolol can cause lymphocyte reduction and dysfunction, and reduced SAP occurrence [20]. Maier et al. found that using β-adrenergic receptor blockers in experimental stroke significantly reduced marginal zone B cells, systemic IgM levels, and occurrence of SAP [21]. Zhang et al. discovered that sympathetic nerve activity significantly increased after stroke by measuring heart rate variability indicators in patients with acute ischemic stroke [22]. Numerous experiments indicate that increased sympathetic excitability after acute stroke mediates an imbalance between pro-inflammatory and anti-inflammatory factor secretion, inhibiting Th1-type immune responses, reducing peripheral immune cells, and suppressing cellular immunity, leading to post-stroke immunosuppression and an increased risk and severity of infections.

##### Hypothalamic–pituitary–adrenal (HPA) axis

Cytokines released after stroke, such as Interleukin (IL)−1, IL-6, IL-10, and TNF-α, activate the HPA axis, leading to excessive glucocorticoid release [23]. Glucocorticoids inhibit the production of pro-inflammatory cytokines, induce anti-inflammatory cytokines, and suppress T cell proliferation and apoptosis. This process correlates with stroke severity and lymphocyte reduction induced by the stroke [24]. Prass et al. found that using glucocorticoid receptor blockers to inhibit the HPA axis can prevent lymphocyte reduction, apoptosis, and monocyte dysfunction [11].

##### Parasympathetic nervous system

Engel et al. found that cutting the parasympathetic nerve or cholinergic activation targets, such as the α7-acetylcholine receptor, increased immune response and prevented stroke-associated pneumonia in a mouse model of middle cerebral artery occlusion [25]. This finding suggests that the parasympathetic nervous system plays a crucial role in stroke-associated infections. When activated, the parasympathetic nervous system reduces the production of pro-inflammatory cytokines (e.g., TNF-α, IL-1β, and IL-18) in immune cells like macrophages through the acetylcholine-α7 nicotinic acetylcholine receptor (α7 nAChR) pathway, leading to local immunosuppression [26, 27].

#### Immune inflammatory response

Stroke can trigger a systemic inflammatory response, including the activation of immune cells, the release of inflammatory cytokines, and the atrophy of immune organs.

##### Microglia

Following ischemic stroke and vascular occlusion, the affected brain tissue experiences immediate local inflammation. During the acute phase of stroke, microglia, the resident immune cells of the central nervous system (comprising about 10% of brain glial cells), are rapidly activated. Additionally, innate immune cells from peripheral blood, such as monocytes and neutrophils, can infiltrate the brain through blood vessels and meninges [28]. Danger-associated molecular patterns released by dying neurons, such as ATP (Adenosine Triphosphate) and HMGB1 (High Mobility Group Box 1), play a crucial role in activating microglia. Once activated, microglia release a range of inflammatory mediators, including cytokines (e.g., tumor necrosis factor-α, interleukin-1β), chemokines, and reactive oxygen species [29]. These mediators attract additional immune cells to the injury site and contribute to the inflammatory response, which can impair the body’s defense against pathogens. Furthermore, microglia also have a regulatory function in the post-stroke inflammatory response, secreting anti-inflammatory cytokines such as interleukin-10 to mitigate excessive inflammation and maintain inflammatory balance [30, 31].

##### Cytokines

Cytokines may regulate the development of the inflammatory response and the activation of immune cells through various mechanisms in the immune inflammation following a stroke. Cytokines interact with cell surface receptors and corresponding signaling pathways, further activating multiple downstream effector molecules, such as nuclear transcription factor NF-κB and MAPK(Mitogen—Activated Protein Kinase). The activation of these signaling pathways can regulate the transcription and translation of inflammation-related genes, promoting the sustained development of the inflammatory response. After a stroke, cytokines are released in the damaged area, such as tumor necrosis factor-α (TNF-α), interleukin-1β (IL-1β), and interleukin-6 (IL-6) [32]. These cytokines can exacerbate the inflammatory response through multiple pathways, promoting the accumulation of immune cells in the damaged area and triggering an inflammatory cascade. Additionally, cytokines can stimulate the activation and proliferation of immune cells (e.g., macrophages, T cells, and B cells), further amplifying and aggravating the inflammatory response. These activated immune cells produce more cytokines, forming a positive feedback mechanism that perpetuates the inflammatory response. However, excessive or prolonged inflammation can worsen brain damage. Therefore, balancing and regulating cytokine release may be an important strategy for managing post-stroke inflammation.

##### Immune organ atrophy

The spleen, the largest lymphatic organ in the body, plays a significant role in the post-stroke immune-inflammatory response process. Following a stroke, a large number of immune cells are rapidly released into the bloodstream and migrate to the damaged brain tissue. This process can lead to a noticeable reduction in spleen size within days or weeks. The spleen's involvement in post-stroke inflammation is multifaceted. It enhances the immune response by promoting the proliferation and activation of immune cells, including T cells, B cells, and macrophages. Additionally, the spleen secretes various cytokines such as TNF-α, IL-1β, and IL-6, which aid in activating inflammatory cells and releasing inflammatory mediators. The spleen also regulates the production and release of vasoactive substances like nitric oxide (NO) and thromboxane A2 (TXA2), influencing vascular tension, blood rheology, and thrombosis, thereby affecting the inflammatory response. Lee et al. [33] used a novel partial MHC II structure, DRm Q, to isolate inflammation in the spleen and reverse spleen atrophy. Understanding the spleen's role in post-stroke immune inflammation can elucidate the complexity of the inflammatory response and provide a foundation for developing therapeutic strategies.

#### Gut-brain axis

The gut-brain axis refers to the interaction and communication between the brain and the gut through neural conduction, hormone release, and immune factors. Under normal conditions, the body maintains a dynamic balance of the gut microbiota through various mechanisms. However, a stroke can quickly disrupt this balance, potentially due to ischemia affecting the gut, which causes shedding and necrosis of intestinal mucosal cells and results in excessive nitrate production. This disruption can impair the intestinal barrier, increasing permeability and susceptibility to bacterial translocation, which raises the risk of post-stroke infections [34, 35]. Research indicates that post-stroke abnormalities in bile acid metabolism may reduce secondary and primary bile acid levels in the cecum, promoting the overgrowth of harmful commensal microbes and heightening the risk of pneumonia in stroke patients. Butyrate, a short-chain fatty acid, can enhance resistance to respiratory pathogens by boosting the antibacterial activity of monocytes and macrophages in vitro. A low abundance of butyrate-producing anaerobic bacteria in the gut is associated with an increased risk of post-stroke infections [36]. Additionally, the activation of the autonomic nervous system after a stroke can influence gut microbiota composition either directly, through the release of neurotransmitters and peptides, or indirectly, via immune inflammation. Studies have shown that over 70% of bacteria found in the stroke patients’ lungs are common gut commensals [37]. Furthermore, research suggests that post-stroke gut dysbiosis can initiate T cell migration, leading to an increase in IL-17 + γδ T cells migrating from the gut to the brain, accompanied by reduced regulatory T cells.

#### Lung-brain axis

The lungs are crucial for respiration, pulmonary circulation, and immune regulation. Research has shown that T cells, which can induce brain autoimmunity, can migrate to lung tissue and persist as memory cells for extended periods. To explore whether lung microbiota influences the immunity of central nervous system, Alexander Flügel et al. employed an experimental model of lung autoimmunity encephalomyelitis in rats. They found that neomycin injection altered the lung microbiota, leading to increased bacterial lipopolysaccharide (LPS) production. Alexander Flügel et al.’s study demonstrated that lung microbiota can influence brain microglia, confirming the existence of the lung-brain axis [38]. Recently, Professor Bryan G. Yipp’s team at the University of Calgary discovered that LPS exposure in lung TRPV1 + (Transient Receptor Potential Vanilloid Subtype 1) sensory neurons via TLR4 (Toll-like Receptor 4) resulted in severe disease, behavioral changes, inflammation, and hypothermia following infections with Pseudomonas aeruginosa and virulent Escherichia coli [39]. However, inflammation alone did not account for the diverse disease manifestations. Instead, bacteria stimulated corticotropin-releasing hormone neurons in the paraventricular nucleus of the hypothalamus via lung nociceptors, inducing acute stress responses and varying disease behaviors. Bryan G. Yipp’s research highlights the role of sensory neurons in regulating disease behavior during lung infections and suggests potential new treatment strategies. Current research on the lung-brain axis's role in post-stroke infections is still emerging. Further exploration of the neural, humoral, and immune-inflammatory regulatory mechanisms involved may provide deeper insights into this complex interaction.

### Risk factors

Age is a significant independent risk factor for stroke. Data show that for every 10-year increase in age among patients with acute stroke, the relative risk of infection rises by 1.24 [40]. Besides age, other well-recognized risk factors include smoking, atrial fibrillation, congestive heart failure, diabetes, and chronic obstructive pulmonary disease [41]. Additionally, stroke severity is closely linked to increased infection risk. In one study, an NIHSS(National Institutes of Health Stroke Scale) score greater than 16 was associated with a 29% and 21.5% increased likelihood of pneumonia in different cohorts [42].Hospitalized stroke patients are also at risk of infection due to invasive procedures such as mechanical ventilation, intravenous catheters, and urinary catheters. Stroke can result in limb paralysis or decreased mobility, which increases the risk of pressure sores. Prolonged immobility can cause pressure damage to the skin and soft tissues, potentially leading to bacterial infections. Furthermore, motor dysfunction may impair urination, leading to urinary retention and an elevated risk of urinary tract infections. Malnutrition, common among stroke patients, can exacerbate their condition and increase mortality [43–46]. Research indicates that malnutrition impairs immune function, reducing the body’s resistance to bacteria and pathogens, and may contribute to increased drug resistance and infection risk. Additionally, the use of immunosuppressive drugs and anticoagulants can indirectly elevate infection risk.

### Differential pathophysiology and clinical features of non-aspiration stroke-associated infections by site

Pulmonary infections are the most common type of NA-SAI. The primary risk factors for pulmonary infections include advanced age, severe stroke (e.g., high NIHSS score), and prolonged bed rest. The pathophysiological basis is mainly related to post-stroke neuroimmune dysregulation, activation of the autonomic nervous system, and the interaction of the lung-brain axis. Patients often present with symptoms such as fever, cough, sputum production, and rapid breathing.

A meta-analysis has shown that the main risk factors for urinary tract infections include female gender, older age, higher modified Rankin Scale score, and post-void residual urine volume > 100 mL [47]. Typical manifestations of urinary tract infections include dysuria, frequency, urgency, suprapbic pain or flank pain, often accompanied by fever, chills, and/or elevated peripheral white blood cell count. Post-stroke bladder dysfunction and early occurrence of urinary retention or incontinence may be important causes of urinary tract infections [48]. On the other hand, invasive procedures such as indwelling catheterization and contamination caused by the catheter itself can directly induce urinary tract infections, with the risk of infection increasing with the duration of catheterization [49]. These factors disrupt the normal defense mechanisms of the urinary tract, increasing the risk of bacterial colonization and infection.

Multi-site infections usually occur in patients with severely compromised immune function and have a more complex pathophysiological basis, involving systemic immune suppression, multi-organ dysfunction, and microbial dysbiosis. The main risk factors for multi-site infections include the presence of multiple underlying diseases (such as diabetes and chronic obstructive pulmonary disease), use of immunosuppressive drugs, and prolonged hospitalization. Patients often present with multi-system symptoms, such as fever, fatigue, dyspnea, and urinary irritation symptoms. Imaging and laboratory examinations may reveal evidence of infections in multiple sites.

### Prevention and treatment strategies

#### Management of risk factors

For patients with ischemic stroke, it is crucial not only to administer routine antiplatelet, circulatory improvement, and lipid-lowering treatments but also to intensify the management of risk factors such as blood pressure and blood sugar and to ensure adequate nutritional intake. Recent clinical studies have systematically analyzed the risk factors for stroke-associated pneumonia (SAP) and have developed several risk prediction scales, including the PANTHERIS score, AIS-APS score, and ISAN score. These scales enable researchers to stratify the risk of SAP in stroke patients and establish predictive models, facilitating individualized management strategies [50]. Our preliminary research has identified independent risk factors for non-aspiration stroke-associated infections (NA-SAI) to include age, female gender, hypertension, and elevated blood urea nitrogen levels [5]. We are currently expanding our sample size to construct a robust predictive model for NA-SAI, which will provide evidence for identifying modifiable risk factors and preventing NA-SAI in clinical practice.

#### Anti-infection treatment

Antibiotic therapy remains the primary method for treating post-stroke infections. However, the effectiveness of antibiotics in preventing infections or improving outcomes remains debated. The STROKE-INF [51] and PASS [52] studies found that empiric antimicrobial prophylaxis did not significantly reduce the incidence of pneumonia or improve the prognosis of stroke patients. Consequently, future research should focus on evaluating the effectiveness of targeted antibiotic therapy.

#### Anti-inflammatory and immunomodulatory treatment

Ongoing research increasingly highlights the potential of targeting anti-inflammatory and immunomodulatory responses to salvage ischemic brain tissue and improve stroke outcomes. Several drugs are currently undergoing clinical trials to modulate specific inflammatory or immune pathways, including the IL-1 receptor antagonist Anakinra, Natalizumab, and immunotherapies related to intravenous thrombolysis [53]. Additionally, enhancing immune function with immunostimulants has shown promise. For instance, Thymosin α1 can activate dendritic cells, Th cells, and NK cells via the TLR pathway, which may be beneficial in managing immunosuppressive conditions [54]. Stem cell transplantation also holds potential for neuroprotection and immunomodulation in ischemic stroke, representing a promising avenue for future treatments.

#### Gut microbiota regulation

Research indicates that increasing probiotics and fecal microbiota transplantation may enhance immune conditions in patients, though the underlying mechanisms and overall effectiveness require further investigation [55]. Animal studies have demonstrated that daily oral administration of probiotic mixtures can reduce the severity of stroke-associated pneumonia in mice and significantly improve neurological deficits [56]. Furthermore, fecal microbiota transplantation from healthy donors has been shown to reduce infarct size and improve stroke outcomes [57]. While these therapeutic approaches are still in the early stages of research, they offer promising new strategies for preventing and treating non-aspiration stroke-associated infections.

#### Multifaceted treatment strategies for NA-SAI

Based on current literature, various therapeutic strategies have shown promising potential. In terms of anti-inflammatory and immunomodulatory therapies, ongoing studies are actively exploring these approaches. For instance, thymosin α1 has been employed in the treatment of COVID-19 patients to improve immune function, particularly in those with reduced T-cell counts [58]. Although these studies have not directly targeted NA-SAI, they offer valuable insights into the immunomodulatory application of thymosin α1. Therefore, thymosin α1 may have potential in treating non-aspiration stroke-associated infections (NA-SAI), however, further dedicated research is needed to confirm its efficacy and safety. Additionally, therapeutic interventions aimed at modulating the gut microbiota, such as probiotic supplementation and fecal microbiota transplantation, have demonstrated favorable outcomes in animal models by mitigating the severity of stroke-associated pneumonia and improving neurological deficits. Nevertheless, more clinical studies are required to validate their effectiveness and safety in humans. Overall, a multi-targeted combination therapy—simultaneously modulating immune responses, inflammation, and the gut microbiota—may represent the future direction for effectively preventing and treating NA-SAI.

Overall, the prevention and treatment strategies for NA-SAI are still in the exploratory phase and lack direct experimental evidence. We stress that future research needs to pay more attention to the specific application and effectiveness of these strategies in NA-SAI to provide stronger support for clinical practice.

## Conclusion

Non-aspiration stroke-associated infection (NA-SAI) remains a frequent and serious complication of stroke, with profound implications for patient prognosis and mortality. Despite its clinical significance, current research on NA-SAI is limited, leaving critical gaps in our understanding of its underlying pathophysiological mechanisms, risk factors, and optimal management approaches. Addressing these gaps is essential not only for enhancing early diagnosis and risk stratification but also for refining prevention and treatment strategies. Animal models have played a crucial role in elucidating the pathophysiological mechanisms of NA-SAI. Through experimental studies, the involvement of the sympathetic nervous system, gut microbiota, and other factors in NA-SAI has been demonstrated. However, there are inherent differences between animal models and humans; differences in physiological structure, immune systems, and living environments mean that findings from animal studies cannot be directly extrapolated to human subjects. Human studies, on the other hand, better reflect real-world clinical scenarios, but are limited by ethical considerations and individual variability, making research more challenging. Future investigations should further integrate animal and human studies—using animal experiments to provide a theoretical foundation and research direction for clinical investigations, while simultaneously validating these findings in human studies—to achieve a more in-depth understanding of NA-SAI and to develop more effective strategies for its clinical prevention and management. Furthermore, multidisciplinary research efforts are needed to elucidate the complex interplay between the immune response, microbiota, and central nervous system in the context of stroke, which may reveal novel therapeutic avenues.

## Data Availability

No datasets were generated or analysed during the current study.
